# Secondary uses and the governance of de-identified data: Lessons from the human genome diversity panel

**DOI:** 10.1186/1472-6939-12-16

**Published:** 2011-09-26

**Authors:** Stephanie M Fullerton, Sandra S-J Lee

**Affiliations:** 1 Department of Bioethics & Humanities, University of Washington School of Medicine, Seattle, WA 98195, USA; 2 Center for Biomedical Ethics and Department of Pediatrics, Stanford University School of Medicine, Palo Alto, CA 94305, USA

## Abstract

**Background:**

Recent changes to regulatory guidance in the US and Europe have complicated oversight of secondary research by rendering most uses of de-identified data exempt from human subjects oversight. To identify the implications of such guidelines for harms to participants and communities, this paper explores the secondary uses of one de-identified DNA sample collection with limited oversight: the Human Genome Diversity Project (HGDP)-Centre d'Etude du Polymorphisme Humain, Fondation Jean Dausset (CEPH) Human Genome Diversity Panel.

**Methods:**

Using a combination of keyword and cited reference search, we identified English-language scientific articles published between 2002 and 2009 that reported analysis of HGDP Diversity Panel samples and/or data. We then reviewed each article to identify the specific research use to which the samples and/or data was applied. Secondary uses were categorized according to the type and kind of research supported by the collection.

**Results:**

A wide variety of secondary uses were identified from 148 peer-reviewed articles. While the vast majority of these uses were consistent with the original intent of the collection, a minority of published reports described research whose primary findings could be regarded as controversial, objectionable, or potentially stigmatizing in their interpretation.

**Conclusions:**

We conclude that potential risks to participants and communities cannot be wholly eliminated by anonymization of individual data and suggest that explicit review of proposed secondary uses, by a Data Access Committee or similar internal oversight body with suitable stakeholder representation, should be a required component of the trustworthy governance of any repository of data or specimens.

## Background

Human tissue and DNA sample collections have proliferated over the last several decades along with increasing secondary use of genotypic and phenotypic data in research. Yet, there is little consensus regarding the effective governance of secondary research uses, beyond adherence to the terms of informed consent. Recent changes to regulatory guidance in the US and Europe [1-3] have further complicated oversight of secondary research by focusing narrowly on risks related to individual identifiability, rendering most uses of anonymized data exempt from human subjects oversight. To identify the implications of such guidelines for harms to participants and communities this paper explores, by way of a case example, the secondary uses of the Human Genome Diversity Project (HGDP)-Centre d'Etude du Polymorphisme Humain, Fondation Jean Dausset (CEPH) Human Genome Diversity Panel, as reported from 2002-2009.

The HGDP-CEPH Human Genome Diversity Panel (hereafter, "HGDP Diversity Panel") is a collection of cultured lymphoblastoid cell lines derived from 1,050 individuals drawn from 51 different human populations [4]. The samples from which the cell lines are derived were collected by multiple independent investigators over a period of years and subsequently donated to the central collection by common agreement [5]. While the collection shares certain similarities (including its name) with the originally proposed [6], and ultimately quite controversial HGDP [7-12], in practice only a minority of its samples were prospectively collected with the intent of contributing to a representative global sample of human genetic variation [5]. Informed consent for academic research use consistent with sample de-identification was verified by HGDP investigators at the time the collection was established in 2002, although the specific terms of consent vary (Ref [4]; Greely, personal communication). Only two other pieces of linked information are archived with samples: (1) the geographic location of sampling (specified by both geographic coordinates and population name), and (2) the sex of the individual from whom the sample was taken. The resource has been described as "useful for SNP [Single Nucleotide Polymorphism] discovery, analyzing SNP and haplotype variability and structure, and for determining global sequence variation at various [genetic] loci" [4].

As an amalgamated set of samples collected by a range of investigators for diverse primary research purposes, the HGDP panel is similar to other recent data sharing initiatives that aim to pool pre-existing samples and/or data to facilitate data mining and different forms of genetic research [13]. However, access to the HGDP Diversity Panel is based not in a formal review of proposed research but rather on an emailed agreement to specified terms of collaboration, which may include a brief description of anticipated research uses (Cann, personal communication). Investigators who obtain aliquots of the collection agree not to transfer DNA samples to other laboratories, to genotype a non-redundant panel of 951 individuals with their chosen genetic markers, and to return those data to the collection's central database at the time of publication (Cann, personal communication). A review of the published research enabled by the resource therefore provides insight into the types of secondary research uses made possible by wide sharing of de-identified data (uses often not available to public inspection), and permits an examination of the potential negative consequences of limited oversight.

## Methods

As of 1 December 2010, DNA from the collection has been distributed to 107 investigators http://www.cephb.fr/en/hgdp/diversity.php Many more researchers have taken advantage of the genotypic (*in silico*) information derived from the sample collection, readily available for downloading either from the CEPH itself or from the website of lead investigators [14]. We identified major classes of secondary research use of the collection from a review of primary scientific articles published between 2002 and 2009 that reported analysis of HGDP Diversity Panel samples and/or data. English-language articles, which cited Cann et al. 2002 (Ref [4], the article that first described the collection) or Rosenberg et al. 2002 (Ref [15], the first major analysis of global genetic variation using the collection), and/or referenced the HGDP in the abstract or title, were identified from the ISI Web of Science^® ^search engine. Although we are confident that most published research using the sample collection or derived data was captured with this search strategy, we cannot be certain that all HGDP Diversity Panel linked publications were included. Our review excluded review articles and articles focused primarily on human versus non-human comparisons, as well as research that had been conducted but not yet published at the time of our search (Spring 2009).

## Results

### HGDP Secondary Uses in the Published Literature

The published analyses of the HGDP Diversity Panel encompass a wide variety of secondary research uses, ranging from analyses of genetic variation aimed at addressing questions of population genetic or medical genetic significance to different forms of methods development (Table 1).

**Table 1:** HGDP Diversity Panel: Classes of Secondary Research Use

Class	Type	Kind	Number	Examples
Analysis	Population Genetic	Characterize Population Variation	48	candidate genes (alcoholism, Parkinson's disease)
				geography (global or regional)
				types of genetic markers (STRs, CNVs)
		
		Infer Human Evolutionary History and/or Ancestry	36	correlation with environment or language
				identification of ancestry informative markers
				patterns of linkage disequilibrium
		
		Infer Effects of Natural Selection	29	genome-wide investigation
				behavioral characteristics (schizophrenia, depression)
				physical characteristics (skin color, brain size)
	
	Medical Genetic	Control Samples for Disease Gene Studies	17	cleft lip and palate
				heroin addiction
				vertebral malformations

Methods	Algorithm Development		14	genetic ancestry estimation
				population structure detection
	
	Molecular Assay		4	multiplex assay development

The vast majority of the publications we identified focused on genetic analyses of variation within and among the different populations represented in the collection (130 of 148 articles total). Forty-eight of these reports characterized genetic variation with respect to geography (global or regional) or described patterns of variation as they were identified for specific candidate genes or distinct classes of genetic markers (e.g. simple tandem repeats, copy number variants). In other cases, assessment of genetic variation was undertaken with the intention of inferring human evolutionary history (36 publications). A further 29 reports described attempts to identify the effects of natural selection on genes previously implicated in behavioral (e.g. schizophrenia, depression) or physical (e.g. skin color, brain size) characteristics, or via a consideration of genome-wide patterns of variation. A smaller number of publications (n = 17) reported using the collection as normal 'control' samples in studies aimed at identifying clinically significant genetic mutations. The remaining 18 reports described methods-oriented research that involved either using HGDP samples to validate new molecular assays or, more typically, using genotype data derived from the collection (e.g. Ref [15]) as the basis for testing algorithms designed to assess aspects of population structure or estimate key demographic parameters.

### Potentially Objectionable HGDP-Related Research

While the vast majority of secondary uses described in these published reports were in line with the original intent of the collection (as described above), a minority of published reports described research whose primary findings could be regarded as controversial, objectionable, or potentially stigmatizing in their interpretation. Because we acknowledge that any assessment in this regard is necessarily subjective (hence ripe for critique and debate), we highlight specific examples only, noting interpretations identified in previously scholarly discussion as problematic.

Table 2 outlines five such examples, including reports for which polymorphisms associated with traits such as addiction, mental illness, or brain size were shown to be differentially distributed with respect to population background, or in which patterns of genetic variation were linked to social identity (e.g., Jewish ancestry) or geographic location. While none of these findings is likely to have *directly *affected the individuals whose samples and/or data were analyzed, to the degree that these reports support potentially unfavorable conclusions about the populations from which participants were drawn, they could be regarded as posing indirect harms to both individuals and groups.

**Table 2:** Findings Supported by the Use of the HGDP Diversity Panel

Type	Publication	Excerpt from Abstract	Interpretation
Addiction	Bierut, L. J., et al. (2008). "Variants in nicotinic receptors and risk for nicotine dependence." Am J Psychiatry 165(9): 1163-71.	"A genetic variant marking an amino acid change showed association with the smoking phenotype (p = 0.007)...t its frequency varied across human populations (0% in African populations to 37% in European populations)."	Europeans are More Susceptible to Nicotine Dependence [29]
Ancestry	Need, A. C., et al. (2009). "A genome-wide genetic signature of Jewish ancestry perfectly separates individuals with and without full Jewish ancestry in a large random sample of European Americans." Genome Biol 10(1): R7.	".. within Americans of European ancestry there is a perfect genetic corollary of Jewish ancestry which, in principle, would permit near perfect genetic inference of Ashkenazi Jewish ancestry."	Jewish People are Genetically Distinct [30]
Genetic Variation	Rosenberg, N. A., et al. (2002). "Genetic structure of human populations." Science 298(5602): 2381-2385.	"...without using prior information about the origins of individuals, we identified six main genetic clusters, five of which correspond to major geographic regions, and subclusters that often correspond to individual populations."	Racial and/or Ethnic Group Differences are "Real" (i.e. Genetic) [31]
Mental Illness	Gardner, M., A., et al. (2006). "Extreme population differences across Neuregulin 1 gene, with implications for association studies." Molecular Psychiatry 11(1): 66-75.	"... allele differences are especially relevant in two SNPs located in a large intron of the gene, as shown by the extreme FST values, which reveal genetic stratification correlated to broad continental areas."	Populations Differ Significantly in Schizophrenia Susceptibility [32]
Natural Selection	Mekel-Bobrov, N., et al. (2005). "Ongoing adaptive evolution of ASPM, a brain size determinant in Homo sapiens." Science 309(5741): 1720-1722.	".. one genetic variant of ASPM in humans arose merely about 5800 years ago and has since swept to high frequency under strong positive selection. These findings... suggest that the human brain is still undergoing rapid adaptive evolution."	Brain Size has Evolved More Rapidly in Non-African Populations [33]

## Discussion

### Secondary Uses of De-Identified Data and the Avoidance of Harm

The Department of Health and Human Services (DHHS) Office of Human Research Protections (OHRP) has deemed that research on specimens or data that have been delinked from personally identifiable information is not subject to federal regulation related to human subjects [2,3], which is consistent with guidelines for exemption by the Common Rule that regulates the protection of human subjects in all federally funded research [16] and the Health Insurance and Portability and Accountability Act (HIPAA) that protects against the disclosure of individually identifiable health information [17]. Neither statute, however, provides clarity on the oversight of secondary use of genetic information which, in sufficient quantity, may - in and of itself - allow re-identification [18,19]. Treating nominally de-identified DNA samples and/or derived genetic information as exempt from human subjects regulation facilitates the goal of data sharing among researchers and institutions while minimizing the potential for harm to individuals arising from public release of confidential personal information [20].

However, harms may emerge when group identification is retained with sample collections, leading to stigmatization or other kinds of "group harm" [21,22]. Individual and group harm may also emerge in the form of a violation of trust when samples are used in research that the original study participants would find objectionable, a form of "dignitary harm" [23]. In 1989, for example, 200 Havasupai tribal members provided blood samples for what was described by researchers at Arizona State University as a population-based study of diabetes. Later, the Tribe discovered that the samples were used in a number of other studies involving research on schizophrenia, inbreeding, and human migration. In 2004, the Tribe filed a lawsuit against the Arizona Board of Regents claiming that the original informed consent agreement was violated by these secondary uses [24]. Under current guidelines, the secondary distribution of individually de-identified data was not subject to research oversight and yet, Tribal research participants (both individually and as a group) experienced harm. Moreover, the harm incurred was not simply due to a "breach of contract" (i.e., uses not specified at the time of consent) but from the use of samples for research purposes regarded as culturally dissonant and deeply objectionable [24]. In 2010, the Board of Regents agreed to pay $700,000 to tribe members as part of a settlement with the Tribe. In addition, the university agreed to return blood samples and provide assistance in building a health clinic on the Havasupai reservation and provide educational scholarships for tribal members [25].

The HGDP Diversity Panel samples are individually de-identified but linked to population of origin and, arguably, certain of the groups represented in the collection have been harmed by findings such as those outlined in Table 2. With respect to the potential for dignitary harm to individuals, there is not enough publicly available information on the terms of informed consent to judge whether the reported research uses are consistent with participants' expectations. Nevertheless, it is not hard to imagine that some contributing participants would regard as objectionable research that attempts to correlate genetic variation with social identity or geographic location, or implies ethnic differences in addiction, mental illness, or intelligence. Indeed, initial objections to the originally proposed Human Genome Diversity Project (which, as noted above, is largely unrelated to the current collection managed by the CEPH) were based in concerns that samples would be used in these and related ways [7-12].

### Implications of HGDP Uses for Research Governance

We acknowledge that the degree to which the research uses described in Table 2 represent a tangible harm to individual research subjects and/or communities is subject to interpretation and disagreement. Our findings are interesting not because of what they say about the secondary uses of the HGDP Diversity Panel *per se*, but because of what they suggest about the range of research uses that are possible when samples and/or data are rendered exempt from research oversight. Investigators and institutions with primary responsibility for standing biospecimen collections and/or data repositories should recognize that potential harms cannot be altogether avoided by removing individually identifying information. While it may be perfectly legitimate, from a narrow regulatory vantage point, to waive research oversight in such cases, foregoing governance of secondary research uses could prove, in certain cases, ethically inadequate [26]. And this will remain true even if all participants have provided explicit permission for broad data sharing and open-ended research use at the time of informed consent.^1^

It is impossible to say whether a more systematic form of oversight on the part of the CEPH, that addressed the potential for group and/or individual dignitary harm, would have avoided these outcomes or resulted in published research better aligned with participants' (presumed) expectations. A challenge for sample collections such as the HGDP Diversity Panel, which have been aggregated over long periods of time, is that original informed consent documents are either unavailable or fail to adequately anticipate the full range of current and potential secondary uses. Hence there is no firm basis to guide a Data Access Committee (DAC) or similar oversight body with respect to whether a proposed use is allowable or prohibited. Moreover, even when consent is available, it is unclear whether this type of front-end review sufficiently addresses the implicit expectations of individual participants or identifies when groups' interests could be significantly compromised by particular classes of investigation.

Rather than grounding decision-making solely in the specifics of the consent language, DACs and similar oversight bodies should consider alternative mechanisms for soliciting the views of individuals with salient insights regarding the interests of participants or their communities. In this way, a more beneficial, and ultimately trustworthy, form of data stewardship will be achieved [27].

## Conclusions

The wide range of published research using the HGDP Diversity Panel demonstrates the utility of a globally-distributed collection of individually de-identified population-based DNA samples and derived genotypic data. Nevertheless, a minority of studies highlighted in our analysis fall into an ethical "grey zone," involving the investigation of research questions which many might regard as socially sensitive and potentially at odds with the desires and expectations of participants. These observations demonstrate that potential risks cannot be wholly eliminated by anonymization and suggest that on-going review of proposed secondary uses will be required for the trustworthy stewardship of even fully de-identified data.

The exact form such review should take is more complex. With the increasing popularity of broad consent, and the intentional re-purposing of older, variably consented, samples and data, consent-led review will be neither sufficient nor flexible enough to safeguard against all possible harms. The current system is ill-equipped to address the potential for group and/or dignitary harms and any decision by reviewers to disallow research that poses such risks may be seen as curbing academic freedom. This tension requires that data stewards adopt procedures that allow due consideration of relevant stakeholder perspectives as part of the review process. This may be achieved either by including participant representatives as voting members of the oversight group tasked with reviewing access requests, by providing participants with periodic updates about the current uses of individual or aggregate data (with the option to withdraw from future research if these uses are not commensurate with their expectations), or by soliciting the perspectives of the original recruiting investigators, who are beholden to participants by dint of on-going research interactions. Simultaneously, the data access review process should be made, as far as feasible, clearly communicated and transparent so that the nature of requests granted and refused are available not just to participants but for wider public inspection and debate. Such approaches will create robust opportunities for identifying and addressing secondary uses that fall into potential ethical grey zones and provide a strong basis for promoting participant and public trust in the broader research enterprise.

## Competing interests

The authors declare that they have no competing interests.

## Authors' contributions

Both authors contributed to the conceptual development of the paper and drafted and revised the manuscript. SSL supervised the identification of published literature and SMF read and classified articles according to reported secondary uses. Both authors have read and approved the final manuscript.

## Endnotes

^1^Recently, the U.S. DHHS and the White House Office for Science & Technology Policy (OSTP) issued an Advance Notice of Proposed Rulemaking (ANPRM) that aims to revise current federal human research protection regulations for the first time since 1991 [28]. The proposed changes acknowledge secondary use of biospecimens and data as potentially identifiable, however only stipulate a requirement of general consent for future use. In addition, the ANPRM proposes expanding the eligibility for secondary use for exemption from human subjects review. Discussion of the unanticipated harms that can emerge from secondary use as illustrated in the case of HGDP Diversity Panel is particularly critical during this period of public comment on the ANPRM.

## Pre-publication history

The pre-publication history for this paper can be accessed here:

http://www.biomedcentral.com/1472-6939/12/16/prepub
